# Bodily confusion: Lower differentiation of emotional and physiological states in student alcohol users

**DOI:** 10.1111/adb.13364

**Published:** 2024-01-26

**Authors:** Aleksandra M. Herman, Marek Wypych, Jarosław Michałowski, Artur Marchewka

**Affiliations:** ^1^ Laboratory of Brain Imaging, Nencki Institute of Experimental Biology Polish Academy of Sciences Warsaw Poland; ^2^ Laboratory of Affective Neuroscience in Poznań, Faculty of Psychology and Law SWPS University of Social Sciences and Humanities Poznań Poland

**Keywords:** alcohol use, alexithymia, AUDIT, body sensation mapping, emBODY, emotions, interoception

## Abstract

**Background:**

Alexithymia, difficulty in recognising and naming emotions, is common among people who use alcohol. There is also emerging evidence that people with alexithymia are unable to distinguish emotions from non‐emotional physiological states. The project aimed to test if alcohol use is related to the way student drinkers experience emotions and physiological states in the body.

**Methods:**

We employed a novel method to study bodily sensations related to emotions and physiological states in the context of alcohol use: the emBODY tool, which allowed participants to mark areas of the body in which they experience various emotions and physiological states.

**Results:**

Students who showed a hazardous pattern of alcohol use (alcohol use disorders identification test [AUDIT] score ≥ 7, N = 91), overall, presented higher alexithymia levels and coloured larger areas for emotions and physiological states (showed less specificity) than those who show low‐risk alcohol consumption (AUDIT ≤ 4, N = 90). Moreover, statistical classifiers distinguished feeling‐specific maps less accurately for hazardous drinkers than low‐risk drinkers [*F*(1,1998) = 441.16; *p* < 0.001], confirming that higher alcohol use is related to higher confusion of emotional and non‐emotional bodily feelings.

**Conclusions:**

Plausibly, this increased bodily confusion drives alcohol consumption: alcohol may serve as a means of dealing with undifferentiated changes in psychophysiological arousal accompanying emotional states.

## Graphical abstract

Students with varying alcohol consumption levels differ in the precision of their bodily representations of emotions and non‐emotional states. Greater alcohol use is associated with increased confusion between emotional and non‐emotional bodily sensations, potentially driving alcohol use as a coping mechanism for undifferentiated arousal.

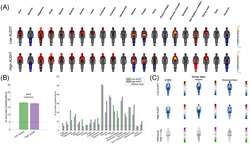

## 1 INTRODUCTION

Alexithymia is a subclinical phenomenon involving a lack of emotional awareness or, more specifically, difficulty in identifying and describing emotions and, crucially, distinguishing them from non‐emotional bodily sensations.^18^ Alexithymia is associated with psychopathology, including anxiety, depression, eating disorders,^34^, ^36^ as well as substance use disorders.^17^ The importance of alexithymia is increasingly recognised and studied in the context of alcohol use and misuse^13^, ^32^, ^38^ as it affects between 30% and 49% of individuals with alcohol dependence,^9^ compared to just about 10% in the general population.^30^ Emerging evidence also suggests that alexithymic traits are higher in student social alcohol drinkers and binge drinkers (in whom repeated episodes of drinking till intoxications are intermitted with episodes of no‐drinking) compared to their low‐drinking counterparts.^5^, ^15^

Alexithymia is also tightly linked to the concept of interoception—the sense of the physiological condition inside one's body.^19^ Alexithymia is increasingly recognised as a general deficit in interoception,^6^, ^11^ which may result in difficulty in discriminating between different emotional and non‐emotional bodily states. Accordingly, higher levels of alexithymia have been linked to more similar perceptions of emotional and non‐emotional states (e.g., hunger being very similar to feeling hot, or sadness to physical fatigue).^6^ Research also indicates that alexithymia, specifically difficulty in identifying feelings, plays a mediating role in the relationship between sensitivity to bodily sensations and alcohol consumption among student social drinkers.^5^ Thus, difficulties in recognising emotional and physiological states may lead to maladaptive responses, such as turning to alcohol to cope with unpleasant sensations.^14^ Furthermore, a reduced ability to detect changes in bodily states, such as failing to sense intoxication, can exacerbate drinking behaviour, resulting in more adverse consequences.^8^ Therefore, it is important to gain a better understanding of alexithymia, specifically the differentiation between emotional and non‐emotional bodily feelings, in the context of alcohol use and problematic drinking at varying levels of alcohol use problems. Such insights may prove valuable in developing innovative treatments or coping strategies for affected individuals.

Currently, one of the most popular methods of assessing alexithymia is the Toronto Alexithymia Scale (TAS),^1^ a 20‐item self‐report questionnaire. One drawback of this measure is that it requires good self‐insight to provide accurate ratings. Additionally, although TAS assesses difficulty in identifying emotions, it does not specifically assess difficulty distinguishing bodily sensations of emotional arousal from other physiological states, providing a need for additional assessment methods.

The interactive online tool, emBODY, is a self‐report measure that allows participants to mark areas of the body in which (and with what intensity) they experience various emotions and other physiological states.^26^, ^27^ Previous work using the emBODY tool has indicated that emotion words, guided emotion imagery, emotional movies and facial expressions produced highly consistent bodily sensation maps (BSMs) within the target emotions.^26^ Importantly, comparable BSMs of emotions have been found across Western and Eastern samples and in several languages, suggesting that this topography of emotional feelings in the body is preserved across different cultures.^26^, ^27^, ^37^ The BSMs approach has also been used to study the topography of emotions in clinical populations, for example, in patients with schizophrenia^35^ and major depressive disorder.^24^ Noteworthy, one study investigated the BSMs of emotions in the context of alexithymia: when compared to non‐alexithymic individuals (TAS total score ≤46) those with probable alexithymia (TAS total score >46) marked significantly smaller areas in the body outlines for 3 out of 17 emotions, specifically happiness, jealousy and fear.^23^ These results may suggest that the probable alexithymia group has a comparatively blunted degree of emotion‐related bodily sensation in some of the emotions. Yet, to our knowledge, no research to date has looked at the differentiation or confusion of emotions and other, non‐emotional physiological states using the body maps approach.

Thus, in the current study, we sought to explore the topographical representation of bodily sensations related to emotions‐ *and* non‐emotional states (further referred to as *physiological states*) in student alcohol users. We focused on student drinkers in particular as alcohol use is widespread in early adulthood and is frequently associated with difficulties in emotional processing.^15^, ^22^ Our a priori hypotheses were three‐fold: (i) high drinkers (as identified with a well‐validated Alcohol Use Disorder Identification Test)^28^ would show higher alexithymia levels (as assessed with TAS) compared to low drinkers; (ii) low and high drinkers would endorse significant differences in the topography and/or extent of different BSMs; and (iii) high drinkers would show higher similarity (greater confusion) between the BSMs of emotions and physiological states than low drinkers.

## 2 METHODS

### 2.1 Participants

Students from a local university were recruited to participate in an online study in exchange for course credits. Participants had to be 18–30 years old and be current alcohol users in order to take part.

### 2.2 Procedures

**FIGURE 1:**
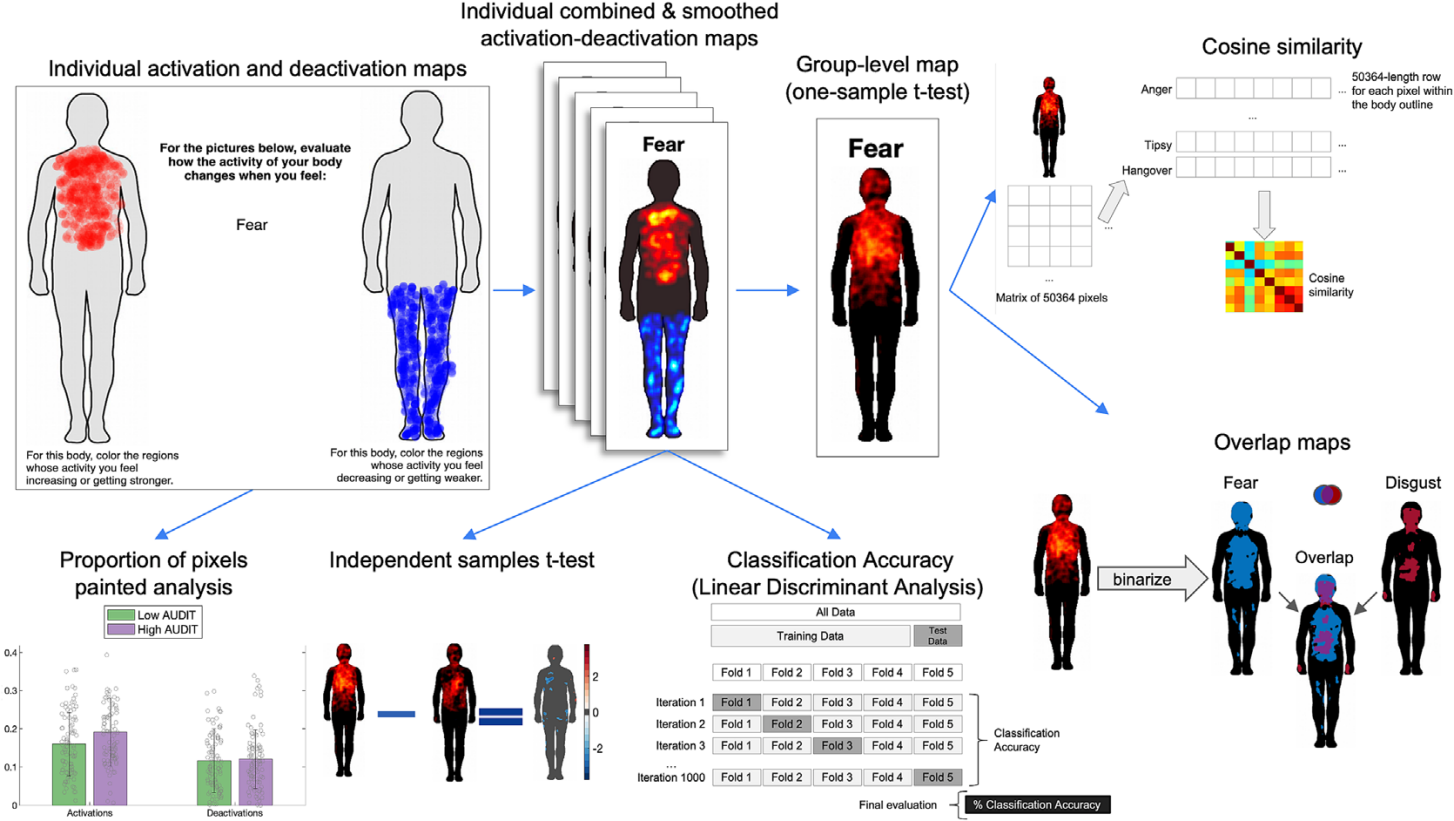
An overview of the body maps experiment and analysis. Participants coloured the initially blank body regions whose activity they felt increasing (left body) and decreasing (right body) during different emotions and non‐emotional states. The proportion of pixels painted (activations and deactivations) was then compared between low‐ and high‐drinking groups. Subjectwise activation–deactivation data were then combined. The obtained individual maps were then subjected to group‐level one‐sample *t*‐test (to provide group‐level body‐sensation maps [BSMs]), independent‐samples *t*‐test (to compare robustness and intensity of sensations between groups) and linear discriminant analysis (LDA; to see whether the BSMs of different states were equally distinctive from each other between the groups). Group‐level maps (resulting from one‐sample *t*‐tests) were further subjected to cosine similarity analysis (comparison of pixelwise similarity between states) and were overlapped, to visualise the topographical similarity between BSMs.

Data collection took place online. Demographic and other self‐report information were gathered using LimeSurvey (https://www.limesurvey.org) while data regarding the topographical representation of emotions and physiological states were acquired using the emBODY tool^26^ following the methodology described previously.^26^, ^27^ Only computers or laptops were an accepted means of study completion. Firstly, volunteers provided informed consent to participate in the study. Next, the task followed (Figure 1). Participants were asked to indicate their somatic experience of 11 emotions, including six basic emotions (fear, anger, disgust, sadness, happiness and surprise), four non‐basic emotions (anxiety, hope, contempt and confusion), and one neutral state as well as 10 non‐emotional physiological states including two specifically alcohol‐related states (tipsy and hangover) and eight other states (nausea, hunger, thirst, physical fatigue, shortness of breath, racing heart, numbness, skin tingling or irritation), yielding 21 items in total, presented in a randomised order. The physiological states were selected based on previous research on the similarity in rating between emotions and physiological sensations in alexithymia.^6^ Participants were shown two human silhouettes on a computer screen with an emotion or a physiological state name placed between the two body outlines. Participants were asked to colour bodily regions whose activity they feel increasing while experiencing a given emotion/state on one body and those whose activity they feel decreasing while experiencing a given emotion/state on the other body outline. Following completion of the body‐mapping task, participants rated on a 10‐point scale the difficulty of the task (where 1: ‘The task was really easy’ and 10: ‘The task was very difficult’). There was no time limit to complete the task but typically it took approximately 15 min. Finally, participants completed questionnaire measures and provided basic demographic information (age and gender).

### 2.3 Questionnaires

The Polish version^31^ of the TAS^1^ is a 20‐item scale used commonly to measure alexithymia. Items are rated using a 5‐point Likert‐type scale (1 = Strongly Disagree to 5 = Strongly Agree). TAS has three subscales: difficulty describing feelings (e.g., ‘It is difficult for me to find the right words for my feelings’), difficulty identifying feelings (e.g., ‘I am often puzzled by sensations in my body’) and externally oriented thinking style (e.g., ‘I prefer talking to people about their daily activities rather than their feelings’). The total TAS score is yielded by summing items. The Polish translation of TAS has demonstrated adequate internal consistency (Cronbach's α = 0.86/0.82, in a non‐clinical sample and alcohol‐dependent sample, respectively).^31^

The Polish version^20^ of the alcohol use disorders identification test (AUDIT)^28^ is a 10‐item questionnaire to screen the frequency and amount of alcohol consumption and alcohol‐related problems (e.g., feeling guilty after drinking) in the past year with a score of 8 out of 40 or more indicating hazardous or harmful alcohol consumption. The AUDIT has demonstrated good internal consistency (Cronbach's α = 0.91).^20^

The Polish version^7^ of the Multidimensional Assessment of Interoceptive Awareness (MAIA)^25^ includes 32 questions assessing interoceptive sensibility. It is used to measure positive and ‘mindful’ attention towards body symptoms (e.g., ‘I feel my body is a safe place’ or ‘I listen for information from my body about my emotional state’). Greater scores indicate higher levels of positive bodily awareness. Internal consistency for MAIA is acceptable with Cronbach's α ranging from 0.66 to 0.82.^25^

The Interoception Sensory Questionnaire (ISQ‐8)^33^ is an eight‐item version of the previously proposed longer version ISQ‐20.^10^ Unlike MAIA, ISQ was developed to assess interoceptive challenges in adults (e.g., ‘I have difficulty making sense of my body's signals unless they are very strong’ or ‘I find it difficult to describe feelings like hunger, thirst, hot or cold’); therefore, higher scores indicate greater interoceptive difficulties. Cronbach's α of ISQ is high (0.96).^33^

### 2.4 Analysis

We explored the topographic representation of each emotion and non‐emotional state across participants following the Nummenmaa and co‐workers' methodology.^26^, ^27^ Specifically, we first reconstructed BSMs from data collected during the web survey. For each participant, a single BSM comprising 50,364 pixels was obtained for each emotion and physiological state, with activations and deactivations coded as positive and negative values, respectively. Responses outside the body outline were masked out. Combined maps were then smoothed using a Gaussian disc (sigma = 5). Next, we screened for sufficient completion rates: participants completing less than 17 (out of 21; i.e., mean—2.5 SDs) of BSMs were removed from further analysis. Moreover, we visually screened all individual maps for anomalous responses (e.g., writing or drawing symbols).

To test our hypothesis that higher problem drinking would be related to (i) higher alexithymia levels, (ii) significant differences across the BSMs and (iii) higher bodily confusion, we separated our sample into three groups based on percentile distribution of AUDIT scores. The top 33 percentile scores were categorised as high drinkers, whereas the bottom 33 percentile scores were categorised as low drinkers. The subsequent analyses compared the demographics, questionnaire and BSMs measures between these two extreme groups using two‐sample *t*‐tests or nonparametric equivalents when necessary. The analyses were performed in MATLAB R2020b and JASP 0.16.2.

For each emotion and state, BSMs were assessed by means of mass univariate *t*‐tests: a one‐sample *t*‐test against zero was performed for each pixel within a BSM for high and low drinkers separately, resulting in a statistical *t*‐map. To account for multiple comparisons, each statistical map was then thresholded using the false discovery rate (FDR) correction (ɑ = 0.05). The FDR correction was also used in all subsequent analyses unless stated otherwise.

#### 2.4.1 Group differences

We first compared the BSMs of each emotion and state between the low‐ and high‐drinker groups with a (pixelwise) two‐sample *t*‐test.

#### 2.4.2 The proportion of pixels painted (PPP)

To further investigate whether any differences between the groups in the BSMs stem from differences in the extent of colouring, we compared the PPP between the two groups. Following past research,^23^ we did that for (unsmoothed) activations and deactivations separately.

#### 2.4.3 Similarity

To quantify the similarity (or confusion) between BSMs of different emotions and states, and (qualitatively) compare it between groups, we performed a pixelwise cosine similarity analysis between each pair of the BSMs for group‐level maps. Similarly to the correlation coefficient, cosine similarity takes into account the intensity and the pattern of painting and results in values from −1 to 1, where −1 indicates complete dissimilarity and 1 complete similarity. However, unlike the correlation coefficient, where the mean value of the map is subtracted from every pixel, the cosine similarity preserves the actual activation/deactivation dimension. See Figure S1 for examples and details.

The corresponding similarity indexes for group‐level BSMs were then subtracted to compare low‐ and high‐drinking groups. This allowed us to (qualitatively) evaluate whether high drinkers indeed report more similar bodily sensations between emotions and states (and which ones) compared to low drinkers.

#### 2.4.4 Overlap

To further visualise the areas of overlap in emotions and physiological states, we used masks made of the significant pixels obtained in the one‐sample *t*‐test analysis for each group. Specifically, for each group, we created binary masks for each emotion and state converting significant and not significant pixels to values of 1 and 0, respectively. Masks were then overlapped on top of each other and added, so the value in a given pixel corresponded to the number of the group‐level BSMs that showed a significant value for a given pixel. Overlap maps were created for each group, and for emotions and physiological states separately, as well as for all BSMs combined. Therefore, these maps allow us to identify commonalities in the pattern of sensations reported across the BSMs at the group level. Additionally, subtraction maps were created to evaluate whether and where in the body high drinkers perceived more common sensations than low drinkers.

#### 2.4.5 Classification

Finally, to test whether different emotions and physiological states are associated with statistically distinct bodily patterns we used statistical pattern recognition with LDA. Following previous research,^16^, ^24^, ^26^ dimensionality was first reduced to 30 components with principal component analysis and then LDA was applied to each group separately. The accuracy of the model was determined with fivefold cross‐validation: data was randomly divided into five subsets, four were used to train the classifier and the fifth was used to test it. This was repeated five times (leaving one subset out each time and training the classifier on the other four). Classifiers were trained to discriminate all stimuli from each other (complete classification).^16^, ^24^, ^26^ To statistically test classifier accuracy against chance level, the cross‐validation was run iteratively 1000 times. Next, the classification accuracy was compared between low and high drinkers with a mixed ANOVA (group by BSM).

For an overview of methods and analyses see Figure 1.

## 3 RESULTS

### 3.1 Participants

Two hundred and thirty‐nine participants met the inclusion criteria and completed the study. Fourteen were excluded due to an insufficient number of BSMs completed, and three more were excluded as a result of a visual inspection of the data. Therefore, 222 individuals were included in the final sample (195 females; age 22.95 ± 3.32; mean number of BSMs painted: 20.34 ± 0.88). Based on percentile distribution, we established grouping cut‐offs: 33rd percentile = 4; and 66th percentile = 7. Thus, those with AUDIT scores ≥7 were categorised as high drinkers (N = 91), while those with AUDIT scores ≤4 were categorised as low drinkers (N = 90). Please note that, since many participants had cut‐off scores (AUDIT = 4, N = 29; AUDIT = 7, N = 18), the sample included in the analysis was larger than two‐thirds of the total number of participants. Groups' characteristics are included in Table 1 and the characteristics of the whole sample are presented in Table S1 (see also Figure S2 for correlations between different measures).

**TABLE 1:** Groups characteristics and comparison.

	Possible range	Low drinkers (AUDIT ≤4; N = 90)		High drinkers (AUDIT ≥7, N = 91)		*t*	df	*p*	Cohen's d
		%/mean	SD	%/mean	SD				
Percentage of females		86.67		90.11		0.65	2	0.723^a^	
Age	18–30	23.34	3.52	22.67	3.30	1.33	179	0.186	0.20
AUDIT score	0–40	2.62	1.18	10.53	3.59	−19.84	179	<0.001	−2.95
Task difficulty	1–10	5.57	2.41	6.30	2.29	−2.09	179	0.038	−0.31
TAS DDF	5–25	10.62	4.24	12.10	4.68	−2.22	179	0.028	−0.33
TAS DIF	7–35	17.03	6.40	19.52	5.83	−2.73	179	0.007	−0.41
TAS EOT	8–40	11.76	4.21	12.45	3.85	−1.16	179	0.248	−0.17
TAS total	20–100	39.41	11.99	44.07	11.31	−2.69	179	0.008	−0.40
MAIA	0–160	98.29	23.59	94.35	23.73	1.12	179	0.264	0.17
ISQ	8–40	15.53	5.50	17.43	6.20	−2.18	179	0.031	−0.32
PPP (overall)	0–1	0.28	0.15	0.31	0.15	−1.59	179	0.113	−0.24

Abbreviations: AUDIT, alcohol use disorder identification test; TAS, Toronto Alexithymia Scale; DDF, difficulty describing feelings; DIF, difficulty identifying feelings; EOT, externally oriented thinking style; MAIA, multidimensional assessment of interoceptive awareness; ISQ, interoception sensory questionnaire; PPP, proportion pixels painted.

^a^ Χ^2^ test with three gender groups: females, males and other.

Groups did not differ in gender distribution or age. As expected by virtue of the percentile split, the high‐drinking group had significantly higher AUDIT scores than the low‐drinking group and that was true for all AUDIT questions (see Supporting Information for details). High drinkers also rated the body‐mapping task as significantly more difficult and showed higher levels of alexithymia (TAS total score), particularly regarding difficulty differentiating feelings and difficulty identifying feelings subscales. There were no group differences in MAIA scores (a measure of ‘positive’ interoceptive sensibility), yet high drinkers reported significantly higher levels of interoceptive difficulties (ISQ score). Overall, these results indicate that our groups were well‐matched in terms of basic demographics and showed expected differences in alexithymia levels and potential interoceptive difficulties.

### 3.2 GROUP DIFFERENCES IN BSMS

**FIGURE 2:**
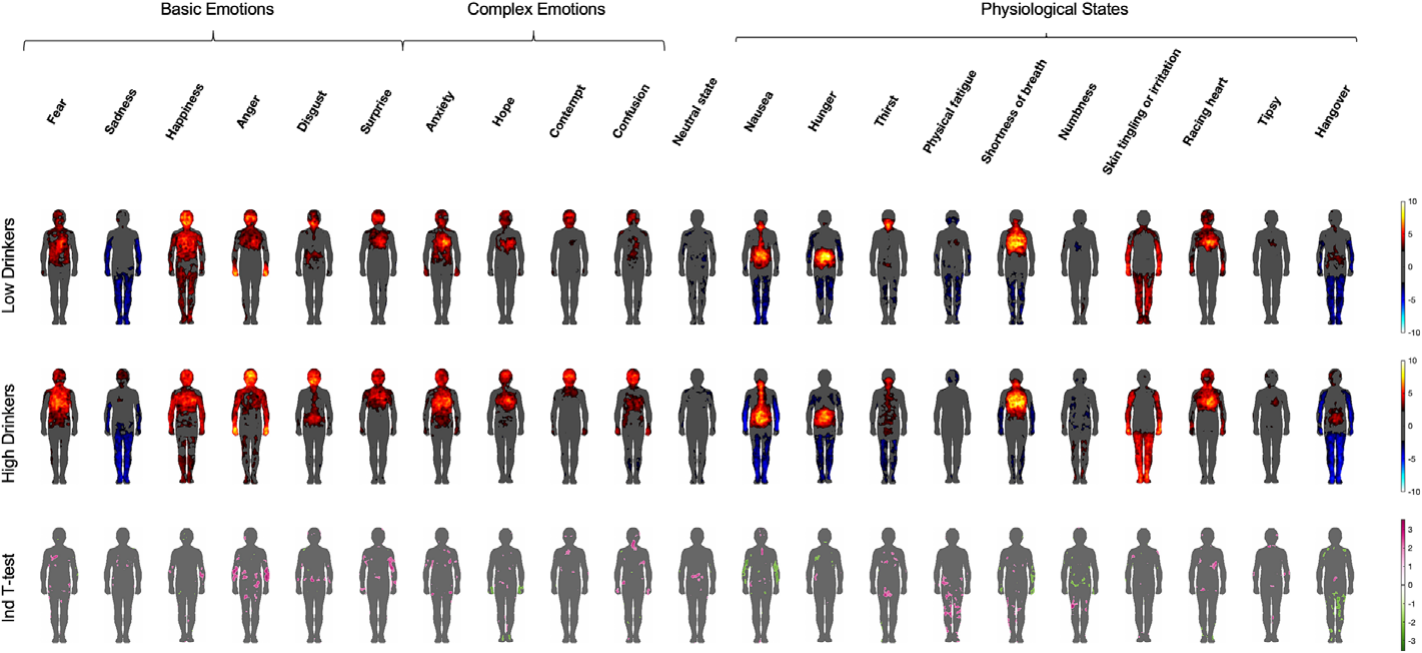
Group‐level bodily sensation maps (BSMs) of emotions and states for low and high drinkers, and the results of the pixelwise independent‐samples *t*‐test (high–low drinkers). Colour bars represent *t*‐values. For the top two panels, warm colours indicate activations and cool colours denote deactivations. For the bottom panel, pinks indicate areas where high drinkers showed significantly more activations than low drinkers and greens represent statistically significant results in the opposite direction.

First, we wanted to visualise the representations of each emotion and physiological state in both groups and inspect any group differences in these representations (see Figure 2 and Figure S4 for standard deviation maps). Visual inspection of the group‐level maps suggests that the topography of BSMs for emotions and states is similar for both groups, yet the BSMs of the high drinkers seem to be larger and more pronounced. This observation is confirmed by the pixelwise independent‐samples *t*‐tests (Figure 2): significant differences were present for every BSM. In the majority of cases, low drinkers showed lower activations than high drinkers, yet, some significant differences in the opposite directions were also present for the BSM of hope, nausea, hunger, shortness of breath, numbness and hangover. Overall, these results suggest stronger and/or less precisely localised representations of emotions and states in high drinkers.

### 3.3 PPP

**FIGURE 3:**
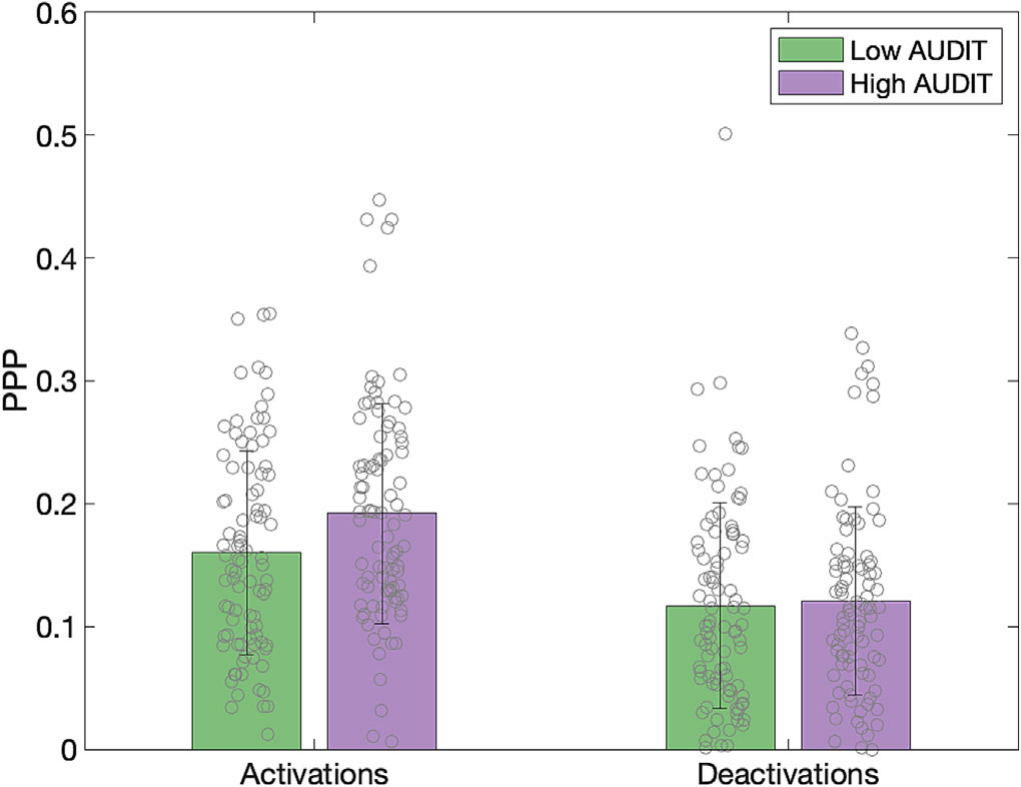
The proportion of pixels painted for activations and deactivations by group. Error bars represent the standard deviation. Grey circles indicate individual data.

To explore whether between‐group differences in BSMs could be attributed to variations in the precision of representations, we conducted an additional analysis using mixed ANOVA to compare the proportion of painted pixels in each BSM between the groups. Since Mauchly's test of sphericity indicated that the assumption of sphericity was violated (*p* < 0.05), the Greenhouse–Geisser correction was applied. This analysis was conducted for all individual BSMs, for activations and deactivations separately (Figures 3 and S5, Table 2) and in both cases showed the main effect of the BSM (e.g., sadness had more localised activations [smaller area painted] than, e.g., fear; *F*(11.83, 2118.23) = 32.78, *p* < 0.001, η^2^ = 0.118; *F*(10.19, 1823.31) = 34.62, *p* < 0.001, η^2^ = 0.127; for activations and deactivation respectively). For activations only, there was also the main effect of group (*F*(1,179) = 6.15, *p* = 0.014, η^2^ = 0.008; for deactivations: *F*(1,179) = 0.11, *p* = 0.745, η^2^ < 0.001), indicating that high drinkers painted more pixels for activations than low drinkers. The group by BSM type interaction did not reach significance either for activations or deactivations (*F*(11.83, 2118.23) = 1.49, *p* = 0.074, η^2^ = 0.005; *F*(10.19, 1823.31) = 0.71, *p* = 0.721, η^2^ = 0.003, respectively). This result shows that, for activations, high drinkers generally coloured in larger areas than low drinkers, which suggests less precise representations of emotions and states in this group.

**TABLE 2:** The results of the mixed ANOVA (classification accuracy ~ group × BSMs) comparing classification accuracy between the groups across all BSMs.

LP	BSMs	Effect	DFn	DFd	F	*p*	Direction
1	Fear	Group	1	1998	1668.68	<0.001	Low > high drinkers
2	Sadness	Group	1	1998	1934.41	<0.001	Low > high drinkers
3	Happiness	Group	1	1998	16,411	<0.001	Low > high drinkers
4	Anger	Group	1	1998	1346.35	<0.001	High > low drinkers
5	Disgust	Group	1	1998	713.52	<0.001	High > low drinkers
6	Surprise	Group	1	1998	8542.39	<0.001	Low > high drinkers
7	Anxiety	Group	1	1998	0.35	0.554	High > low drinkers
8	Hope	Group	1	1998	1597.03	<0.001	High > low drinkers
9	Contempt	Group	1	1998	1026.78	<0.001	High > low drinkers
10	Confusion	Group	1	1998	6298.68	<0.001	Low > high drinkers
11	Neutral state	Group	1	1998	33.54	<0.001	Low > high drinkers
12	Nausea	Group	1	1998	17351.36	<0.001	High > low drinkers
13	Hunger	Group	1	1998	299.75	<0.001	Low > high drinkers
14	Thirst	Group	1	1998	3473.75	<0.001	Low > high drinkers
15	Physical fatigue	Group	1	1998	5448.79	<0.001	Low > high drinkers
16	Shortness of breath	Group	1	1998	1861.61	<0.001	High > low drinkers
17	Numbness	Group	1	1998	1181.62	<0.001	Low > high drinkers
18	Skin tingling or irritation	Group	1	1998	1.75	0.186	Low > high drinkers
19	Racing heart	Group	1	1998	8706.73	<0.001	High > low drinkers
20	Tipsy	Group	1	1998	7.85	0.005	Low > high drinkers
21	Hangover	Group	1	1998	1953.12	<0.001	High > low drinkers

Abbreviation: BSM, bodily sensation maps.

### 3.4 Similarity

**FIGURE 4:**
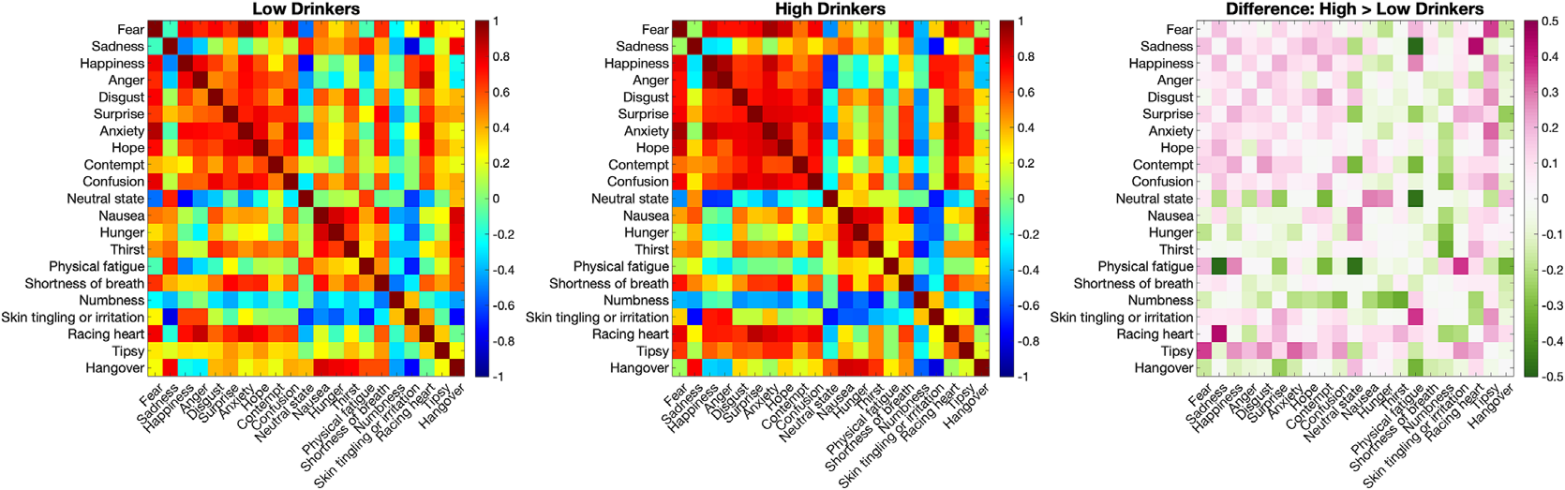
The similarity of group‐level bodily maps (cosine similarity) for each pair of bodily sensation maps (BSMs) for low and high drinkers as well as the difference between the groups.

Next, we checked how similar the representations of different emotions and states within each group are. To quantify the similarity (or confusion) between BSMs we performed a pixelwise cosine similarity analysis between each pair of group‐level BSMs and compared the groups. Figure 4 shows cosine‐similarity matrixes for low and high drinkers, as well as the differences between the groups. Overall, the pattern of similarities between the groups seems to be preserved. Yet, importantly, compared to low drinkers, high drinkers had greater cosine similarities of BSMs of emotions and also between emotions and tipsy state (see Figure 4). This result suggests higher confusion within emotional representations in the high AUDIT group.

### 3.5 Overlap

**FIGURE 5:**
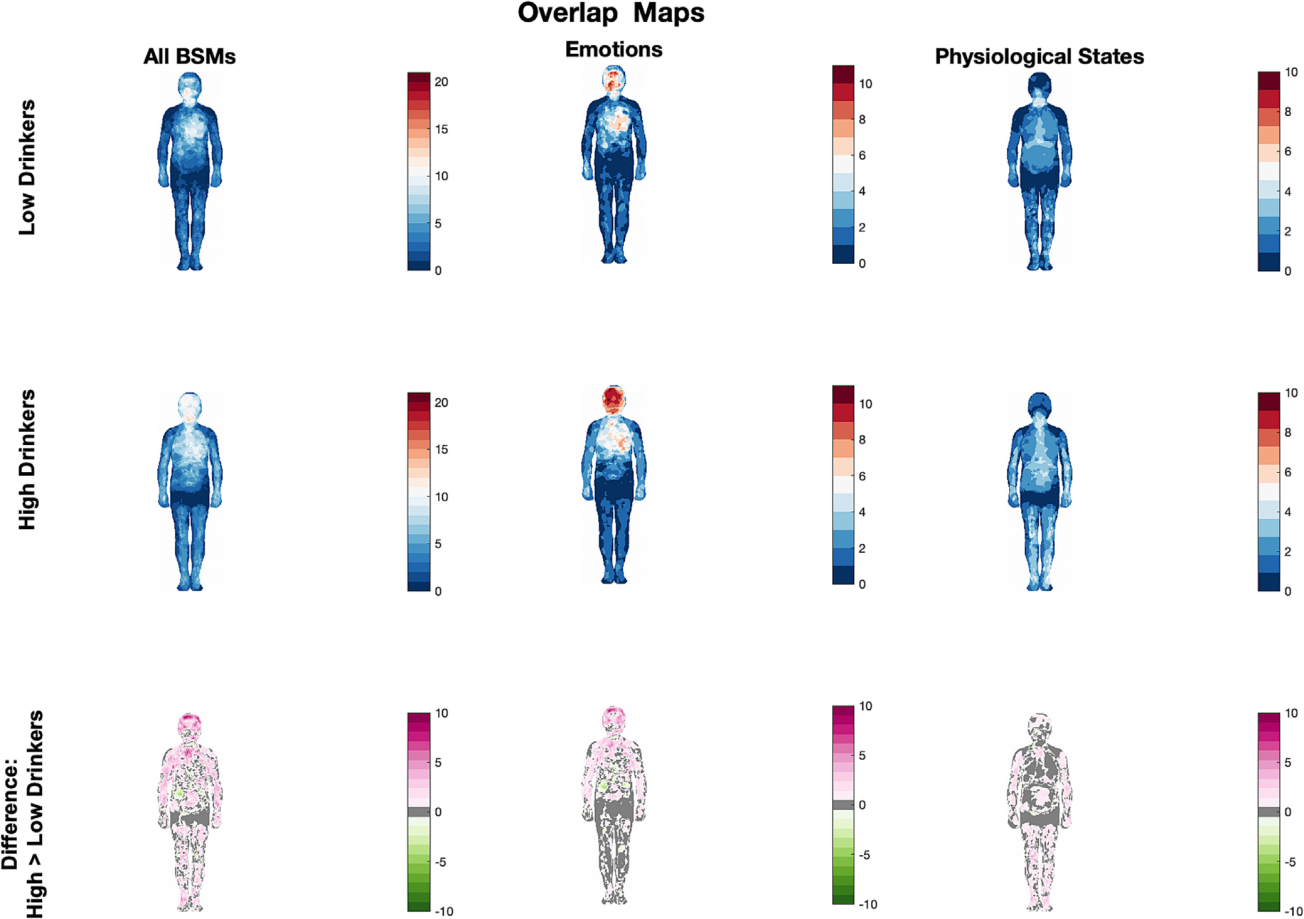
Overlap of group‐level bodily sensation maps (BSMs) for low drinkers (top row), high drinkers (middle row) and the difference between the two (bottom row). The BSMs are overlapped for all BSMs (left column), BSMs of emotions and the neutral state only (middle column), and non‐emotional states (right column). For the top two rows, colour bars represent the number of group‐level BSMs that showed overlapping topography of sensations with warmer colours representing more overlapping BSMs. For the bottom row, the colour bars represent areas where high drinkers showed more/fewer BSMs overlapping than low drinkers.

To gain a deeper understanding of the observed group differences in BSM similarity (or confusion), we analysed the spatial overlap of different emotions and sensations in each group by producing group‐level overlap maps (Figure 5). The maps suggest that high drinkers tend to experience more sensations in the areas of head and chest compared to low drinkers. This is particularly visible for BSMs of emotions for which 10 out of 11 emotions are experienced in the head in high drinkers. This overlap is less visible in low drinkers, who present smaller areas of overlap, which suggests greater spatial differentiation between distinct emotions, which could lead to a better ability to distinguish and name various emotions.

### 3.6 Classification

**FIGURE 6:**
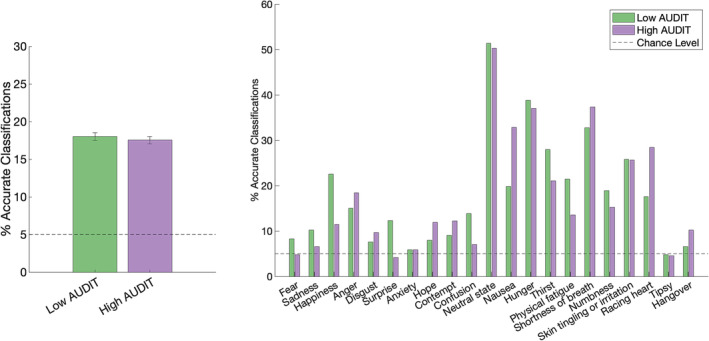
Comparison of classification accuracies (in percent) with complete classification schema between the groups. Overall classification accuracy was averaged across the bodily sensation maps (BSMs; left) and presented separately for each BSM (right). Error bars represent the standard deviation (SD) of the mean (not visible on the right panel as SDs are very small: <0.06%).

Finally, for each group, we wanted to determine if it is possible to distinguish particular emotions and states based on their representations. Thus, we conducted LDA to check whether BSMs for emotions and states are associated with statistically distinct bodily patterns in both groups. The overall classification accuracy (across all of the BSMs) was above the chance level (100/21 = 5%) in both groups, yet for low drinkers, the LDA showed significantly higher classification accuracy (18.01% ± 0.50) than for high drinkers (17.53% ± 0.50; *F*(1,1998) = 441.16; *p* < 0.001; Figure 6). By looking at the accuracy of classification for distinct maps by group (Figure 6), it appears that for low drinkers, all BSMs, but tipsy state, were classified with an accuracy above the chance level, while for high drinkers, BSMs of fear, surprise and tipsy state were classified with below chance‐level accuracies. There was a significant group by BSM interaction (*F*(14.97, 29912.73) = 3585.27; *p* < .001, Table 2), whereby classification accuracy for low drinkers was significantly higher for several BSMs (fear, sadness, happiness, surprise, confusion, neutral state, hunger, thirst, physical fatigue, numbness and tipsy state; for the full confusion matrices, see Figure S6). Nevertheless, for some BSMs (notably anger, disgust, hope, contempt, nausea, shortness of breath, racing heart and hangover), classification accuracy was superior for high drinkers. Thus, overall, these results suggest that high drinkers tend to show statistically unique patterns of sensations for both emotions and states, yet overall show higher confusion between different sensations when compared to low drinkers.

## 4 DISCUSSION

We comprehensively assessed the BSMs of emotions and physiological states experienced by low‐ and high‐student drinkers as a new approach to studying differentiation between (and within) emotional and non‐emotional states associated with alexithymia—an increasingly recognised feature of alcohol use and misuse. We tested three hypotheses, namely, that (i) high drinkers would show higher alexithymia levels compared to low drinkers (as assessed with TAS); (ii) low and high drinkers would endorse significant differences in the topography and/or extent of different BSMs; and (iii) high drinkers would show higher similarity (greater confusion) between the BSMs of emotions and physiological states than low drinkers. Overall, in line with our a priori hypothesis (i), participants classified as high drinkers (AUDIT ≥7) showed higher alexithymia levels than low drinkers (AUDIT ≤4). High drinkers also presented significantly more widespread activations throughout the body related to different emotions and states that were lacking in specificity (ii). Finally, several measures (i.e., similarity analysis, overlaps, LDA) indicated that high drinkers presented lower bodily differentiation between various emotions and physiological states (iii).

Regarding questionnaire findings, high drinkers presented higher alexithymia levels (difficulty differentiating and interpreting feelings subscales) than low drinkers. They also found the body sensations mapping more difficult and reported higher levels of interoceptive difficulties (as measured with ISQ). These differences were present even though both groups were well‐matched in terms of demographics. Interestingly, both groups did not differ in their reports on the MAIA questionnaire, which assesses positive and ‘mindful’ attention towards daily body symptoms. These results suggest that high drinkers in our sample attended to daily bodily feelings equally well or often as low drinkers, but had subjective difficulties interpreting and naming these bodily sensations.

Questionnaire findings align well with our body sensation mapping results. Specifically, our comprehensive assessment of the representation of bodily sensation in student drinkers revealed that high drinkers generally presented a more widespread pattern of sensation that was less differentiated than in low drinkers. Firstly, group differences were present for all 21 bodily maps of emotions and physiological states studied. Secondly, high drinkers, on average, painted more pixels for activations generally. Thirdly, the BSMs of emotions in high drinkers were more similar to each other (had higher cosine similarity) than in low drinkers. This was also evidenced by the overlap of the group level maps in high drinkers, which, indeed, showed that students who presented more problem drinking experienced bodily feelings, particularly emotions, in the very same locations, focused mostly on the head and chest. Finally, the LDA showed that the classification accuracy was overall statistically significantly lower (i.e., lower discriminability between the BSMs) for high drinkers than for low drinkers. Overall, this pattern of results is consistent with previous reports of the undifferentiated BSMs of individuals with probable alexithymia^23^ and autism.^29^ Our findings may point towards the underlying role of alexithymia in the bodily representation of emotional and non‐emotional bodily states in high‐drinking students.

Experiencing a diffused rather than localised pattern of bodily activations and deactivations in high drinkers may hinder the ability to discriminate between distinct states. Indeed, bodily sensations in high drinkers lacked specificity, particularly for the BSMs of emotions, suggesting an indistinct, and possibly confusing, pattern of sensory experience that contrasted with the specific sensations presented by low drinkers. These findings are in line with past research suggesting that alexithymia may be characterised by a general deficit in interoception.^6^ Interestingly, in the current investigation, we also showed a difference in (self‐reported) interoceptive skills between high and low drinkers: high drinkers reported elevated levels of interoceptive difficulties (as measured with ISQ): challenges in reading and interpreting internal bodily signals. This finding aligns further with our results using the emBODY tool. The similarity analysis revealed that, at the group level, high drinkers tended to show an increased similarity between several BSMs, particularly between emotions and emotions and tipsy state. Confusing different emotions or emotions and non‐emotional states with each other is likely to have a variety of negative consequences for one's physical and mental health, as this may reduce one's ability to respond appropriately to their current state. Moreover, difficulty discriminating between different emotions and physiological states may lead to using alcohol as a maladaptive means to deal with undifferentiated arousal.^12^, ^14^ Considering that some young people drink to cope with emotions, which is linked to alcohol‐related problems,^21^ improving the ability to identify one's precise emotional state, might lead to adaptive coping strategies, particularly in students with alcohol‐related issues. Thus, our findings add to a growing body of evidence suggesting that alexithymia is an expression of impaired processing of bodily sensations including physiological arousal, which underpins the development of maladaptive coping strategies, including alcohol use.^5^, ^8^

According to the theory of constructed emotion,^3^, ^4^ our knowledge about emotions is individually constructed based on different information (i.e., through direct bodily experiences, vicarious learning and/or understanding where they are conventionally felt in the body). If people have trouble reading bodily sensations, this knowledge may be less precise, their emotional competencies less beneficial and their coping mechanisms dysfunctional (such as problem drinking). Increasing the level of specificity that characterises verbal representations of an affective experience should lead to a reduction of these dysfunctions.^2^, ^4^ However, this would require an appropriate diagnosis of bodily awareness, which is difficult using verbal reports due to the constructive character of emotional experience as well as the lack of a reliable point of reference in evaluating bodily sensations and socially compatible ways to communicate them. We suggest that BSMs can be especially useful in diagnosing the level of bodily awareness, especially in individuals with increased bodily confusion, including those struggling with alcohol/substance use problems.

Noteworthy, our study was conducted on a group of university students of whom the majority were female. In the future, it would be important to replicate these findings in a more balanced sample of student drinkers and also in the general (non‐student) population. We also did not collect other health‐related information that could potentially influence the results such as a history of other substance use or mental health information. The study was conducted online, limiting our knowledge of the recency of alcohol use or use at the time of study completion. Potentially, acute alcohol intoxication or withdrawal could affect the emBODY task performance. Additionally, as in the current sample, we observed rather subtle differences in terms of confusion of different bodily states; it would also be interesting to conduct a similar study with alcohol‐dependent individuals and a matched control sample, to see to what extent these patterns are preserved/deepened in AUD.

## 5 CONCLUSIONS

We employed the emBODY tool as a novel and implicit method to study alexithymia‐related difficulties discriminating between emotions and physiological states in a group of student drinkers. Students presenting a more harmful pattern of alcohol use presented higher alexithymia levels and higher bodily confusion as shown by our comprehensive analysis of BSMs of emotions and physiological states. The high drinkers differed from low drinkers not so much in the topography of experienced sensations, but more so when it comes to the extent of these sensations and, related, a higher degree of overlap between distinct maps as well as a lower level of ‘uniqueness’ of these topographical maps (as shown by LDA). Experiencing a diffused pattern of bodily activation and deactivation may impede the ability to discriminate between distinct states more readily in high drinkers. Plausibly this lack of sufficient discrimination of bodily sensation and thus the ability to read bodily signals can act as a potential mechanism to continue drinking.

## AUTHOR CONTRIBUTIONS

**Aleksandra M. Herman**: Conceptualization; data curation; formal analysis; investigation; methodology; project administration; resources; software; visualisation; writing—original draft; writing—reviewing and editing. **Marek Wypych**: Methodology; visualisation; writing—reviewing and editing. **Jarosław Michałowski**: Securing ethical approval; project administration; writing—reviewing and editing. **Artur Marchewka**: Supervision; writing—reviewing and editing.

## CONFLICT OF INTEREST STATEMENT

The authors have no competing interests to report.

## ETHICS STATEMENT

The Ethical Committee of the Faculty of Psychology and Law in Poznań at SWPS University approved the study procedures (approval no. 2021‐108). The study was conducted following the Declaration of Helsinki.

## Supporting information


**Figure S1.** Comparison of Spearman's correlation coefficient and cosine similarity (CS) as indexes of similarity between the BSMs. The two indexes have been computed for low drinkers' group‐level BSMs.
**Table S1.** Descriptive Statistics for the whole sample.
**Figure S2.** Correlations (Spearman's correlation coefficient) between self‐reported measures and the averaged proportion of pixels painted in the emBODY task, calculated for the whole sample (N = 222). AUDIT ‐ Alcohol Use Disorder Identification Test, TAS – Toronto Alexithymia Scale, DDF ‐ difficulty describing feelings, DIF ‐ difficulty identifying feelings, EOT ‐ externally oriented thinking style, MAIA ‐ Multidimensional Assessment of Interoceptive Awareness, ISQ ‐ Interoception Sensory Questionnaire, PPP – Proportion Pixels Painted (Overall). * p < .05, ** p < .01, *** p < .001.
**Table S2.** A comparison of alcohol‐related items between the groups.
**Figure S3.** Histograms of responses to the alcohol‐related questionnaire for each group. The questions asked were as follows:
Does your immediate family (parents, siblings) have a history of alcohol disorder (alcoholism)?
**AUDIT 1:** How often do you have a drink containing alcohol?
**AUDIT 2:** How many standard drinks containing alcohol do you have on a typical day when drinking?
**AUDIT 3:** How often do you have six or more drinks on one occasion?
**AUDIT 4:** How often have you found that you were not able to stop drinking once you had started?
**AUDIT 5:** How often have you failed to do what was normally expected of you because of drinking?
**AUDIT 6:** How often have you needed a drink in the morning to get yourself going after a heavy drinking session?
**AUDIT 7:** How often have you had a feeling of guilt or remorse after drinking?
**AUDIT 8:** How often have you been unable to remember what happened the night before because you had been drinking?
**AUDIT 9:** Have you or someone else been injured as a result of your drinking?
**AUDIT 10:** Has a relative or friend doctor or other health worker been concerned about your drinking or suggested you cut down?
**Figure S4.** Standard deviation maps for the bodily sensations of Low and High Drinkers.
**Figure S5.** The proportion of pixels painted for each BSM and group. Error bars represent the standard error of the mean.
**Figure S6.** Confusion matrices for the complete classification scheme for low and high drinking groups between predicted and true classifications. Classifications below the chance level (5%) are depicted in white. Colour bars represent classification accuracies in %.

## Data Availability

Data and analysis code are available via OSF: https://osf.io/qjtp3/?view_only=6359904661ff490c918cd1ad7b6a28c3.
