# Supplementary material for: Bodily confusion: Lower differentiation of emotional and physiological states in student alcohol users
Source: Addict Biol. 2024 Jan 26;29(2):e13364. doi: 10.1111/adb.13364 (PMC10898845; doi:10.1111/adb.13364)
Supplement: Supplementary file 1 — Figure S1. Comparison of Spearman's correlation coefficient and cosine similarity (CS) as indexes of similarity between the BSMs. The two indexes have been computed for low drinkers' group‐level BSMs. Table S1. Descriptive Statistics for the whole sample. Figure S2. Correlations (Spearman's correlation coefficient) between self‐reported measures and the averaged proportion of pixels painted in the emBODY task, calculated for the whole sample (N = 222). AUDIT ‐ Alcohol Use Disorder Identification Test, TAS – Toronto Alexithymia Scale, DDF ‐ difficulty describing feelings, DIF ‐ difficulty identifying feelings, EOT ‐ externally oriented thinking style, MAIA ‐ Multidimensional Assessment of Interoceptive Awareness, ISQ ‐ Interoception Sensory Questionnaire, PPP – Proportion Pixels Painted (Overall). * p < .05, ** p < .01, *** p < .001. Table S2. A comparison of alcohol‐related items between the groups. Figure S3. Histograms of responses to the alcohol‐related questionnaire for each group. The questions asked were as follows: Does your immediate family (parents, siblings) have a history of alcohol disorder (alcoholism)? AUDIT 1: How often do you have a drink containing alcohol? AUDIT 2: How many standard drinks containing alcohol do you have on a typical day when drinking? AUDIT 3: How often do you have six or more drinks on one occasion? AUDIT 4: How often have you found that you were not able to stop drinking once you had started? AUDIT 5: How often have you failed to do what was normally expected of you because of drinking? AUDIT 6: How often have you needed a drink in the morning to get yourself going after a heavy drinking session? AUDIT 7: How often have you had a feeling of guilt or remorse after drinking? AUDIT 8: How often have you been unable to remember what happened the night before because you had been drinking? AUDIT 9: Have you or someone else been injured as a result of your drinking? AUDIT 10: Has a relative or friend doctor or other health worker been [file ADB-29-e13364-s001.pdf]

SUPPLEMENTARY MATERIALS:

Bodily Confusion: Lower Differentiation of Emotional and Physiological States in Student Alcohol Users

Aleksandra M. Herman, Marek Wypych, Jarosław Michałowski, Artur Marchewka

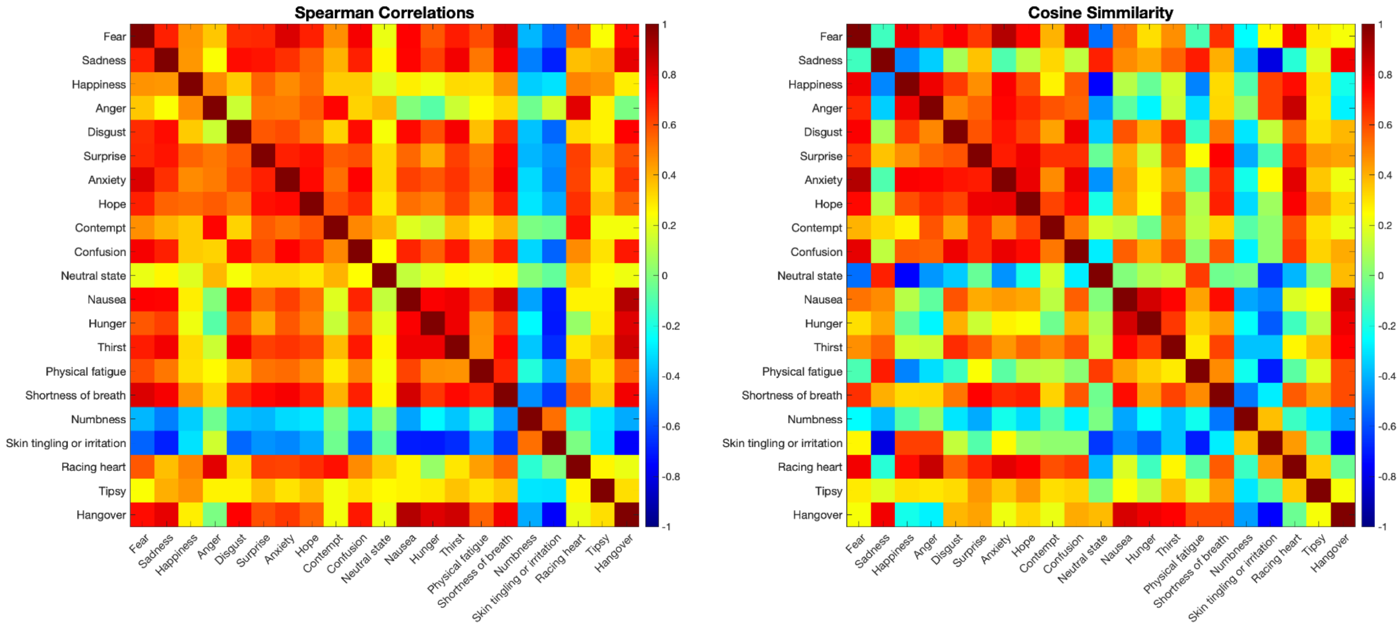

Fig S1. Comparison of Spearman's correlation coefficient and cosine similarity (CS) as indexes of similarity between the BSMs. The two indexes have been computed for low drinkers' group-level BSMs.
In case of BSMs that cover overlapping body areas both correlation coefficient and cosine similarity would result in similar outcomes. For the same direction of sensation, that is both BSMs with activations or both deactivations (e.g. fear and anxiety), both measures result in positive values. Similarly, for the BSMs that cover overlapping body areas showing the opposite direction of sensation (i.e. activation and deactivation e.g. skin-tingling and sadness), both measures result in negative values.
For non-overlapping body areas however (e.g. sadness and disgust) the cosine-similarity results in values close to zero while the correlation coefficient due to subtracting the mean values from all pixels results in non-zero values (positive in the example of sadness and disgust). Thus we believe that cosine similarity values are more intuitive and easier to interpret.

Variable	Valid	Missing	Median	Mean	Std. Deviation	Variance	Range	Min	Max
Task Difficulty	222	0	6.0	6.06	2.38	5.65	9	1	10
DDF	222	0	10.5	11.41	4.64	21.51	20	4	24
DIF	222	0	18.0	18.45	6.30	39.65	28	7	35
EOT	222	0	12.0	12.19	3.95	15.57	17	5	22
TAS	222	0	41.0	42.04	12.07	145.64	59	19	78
MAIA	222	0	95.5	96.42	23.52	553.09	115	34	149
ISQ	222	0	16.0	16.57	5.86	34.36	30	8	38
AUDIT	222	0	5.0	6.37	4.35	18.89	23	1	24

Table S1. Descriptive Statistics for the whole sample.

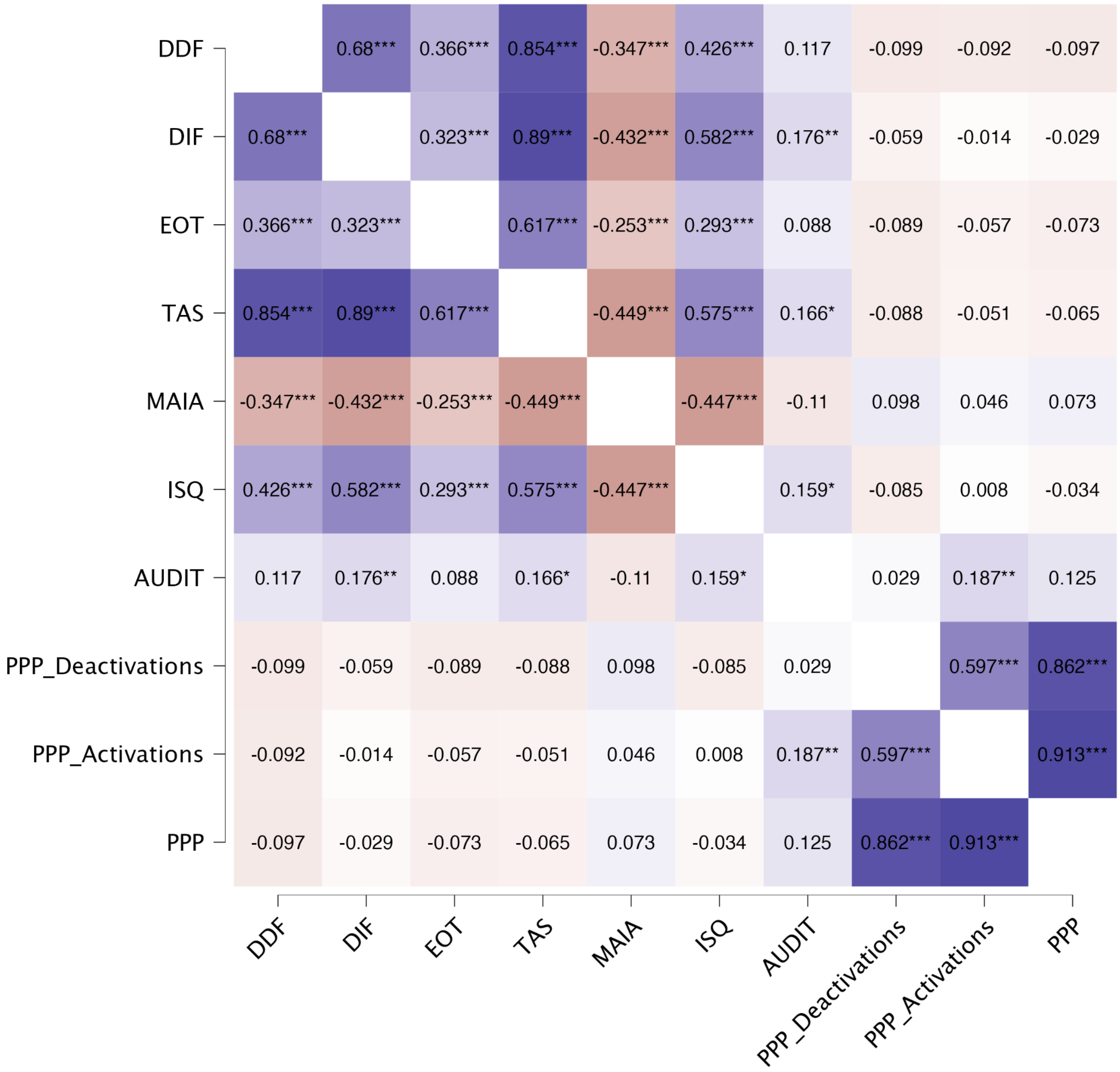

Fig S2. Correlations (Spearman’s correlation coefficient) between self-reported measures and the averaged proportion of pixels painted in the emBODY task, calculated for the whole sample (N = 222). AUDIT - Alcohol Use Disorder Identification Test, TAS - Toronto Alexithymia Scale, DDF - difficulty describing feelings, DIF - difficulty identifying feelings, EOT - externally oriented thinking style, MAIA - Multidimensional Assessment of Interoceptive Awareness, ISQ - Interoception Sensory Questionnaire, PPP - Proportion Pixels Painted (Overall). * $p < .05$, ** $p < .01$, *** $p < .001$.

In line with the group results reported in the main text, higher AUDIT scores were associated with increased alexithymia (particularly difficulty identifying feelings subscale) and interoceptive difficulties (ISQ). At the same time, AUDIT correlated negatively with positive interoceptive awareness (MAIA). Higher AUDIT scores were also linked to a higher proportion of pixels painted as activations in the emBODY task, indicating that students presenting more problematic patterns of alcohol use coloured in larger areas activations for different emotions and states. There were no significant correlations between indexes of alexithymia or interoception and pixels painted.

Welch's t-test					Low Drinkers						High Drinkers					
**Variable**	**t**	**df**	**p**	**Cohen's d**	**Valid**	**Missing**	**Median**	**Variance**	**Min**	**Max**	**Valid**	**Missing**	**Median**	**Variance**	**Min**	**Max**
Familial history of AUD	7.73[a]	3	0.193													
AUDIT1	-10.27	152.85	< .001	-1.53	90	0	1	0.23	1	3	91	0	2	0.57	1	4
AUDIT2	-11.45	155.10	< .001	-1.70	90	0	0	0.39	0	2	91	0	2	0.91	0	4
AUDIT3	-13.20	145.89	< .001	-1.96	90	0	0	0.24	0	1	91	0	2	0.70	0	4
AUDIT4	-5.94	96.57	< .001	-0.88	90	0	0	0.02	0	1	91	0	0	0.61	0	3
AUDIT5	-9.69	101.55	< .001	-1.44	90	0	0	0.02	0	1	91	0	1	0.35	0	2
AUDIT6	-3.98	96.23	< .001	-0.59	90	0	0	0.01	0	1	91	0	0	0.32	0	3
AUDIT7	-11.62	134.26	< .001	-1.73	90	0	0	0.15	0	1	91	0	1	0.57	0	3
AUDIT8	-9.32	125.54	< .001	-1.38	90	0	0	0.08	0	1	91	0	1	0.40	0	3
AUDIT9	-5.20	101.99	< .001	-0.77	90	0	0	0.13	0	2	91	0	0	1.97	0	4
AUDIT10	-3.16	110.55	0.002	-0.47	90	0	0	0.13	0	2	91	0	0	1.14	0	4

[a]Chi-Squared test

Table S2. A comparison of alcohol-related items between the groups.

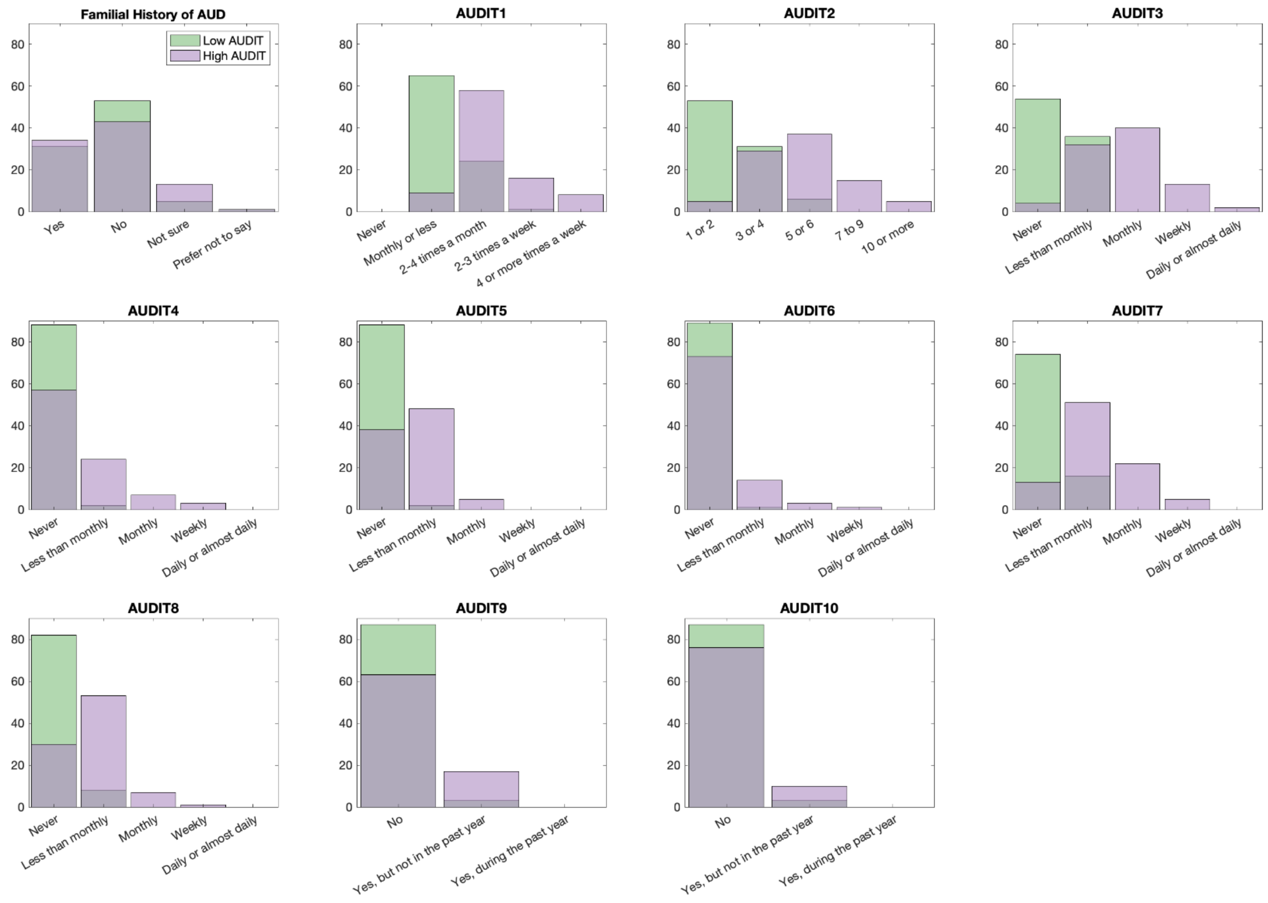

Fig S3. Histograms of responses to the alcohol-related questionnaire for each group. The questions asked were as follows:

Does your immediate family (parents, siblings) have a history of alcohol disorder (alcoholism)?

AUDIT 1: How often do you have a drink containing alcohol?

AUDIT 2: How many standard drinks containing alcohol do you have on a typical day when drinking?

AUDIT 3: How often do you have six or more drinks on one occasion?

AUDIT 4: How often have you found that you were not able to stop drinking once you had started?

AUDIT 5: How often have you failed to do what was normally expected of you because of drinking?

AUDIT 6: How often have you needed a drink in the morning to get yourself going after a heavy drinking session?

AUDIT 7: How often have you had a feeling of guilt or remorse after drinking?

AUDIT 8: How often have you been unable to remember what happened the night before because you had been drinking?

AUDIT 9: Have you or someone else been injured as a result of your drinking?

AUDIT 10: Has a relative or friend doctor or other health worker been concerned about your drinking or suggested you cut down?

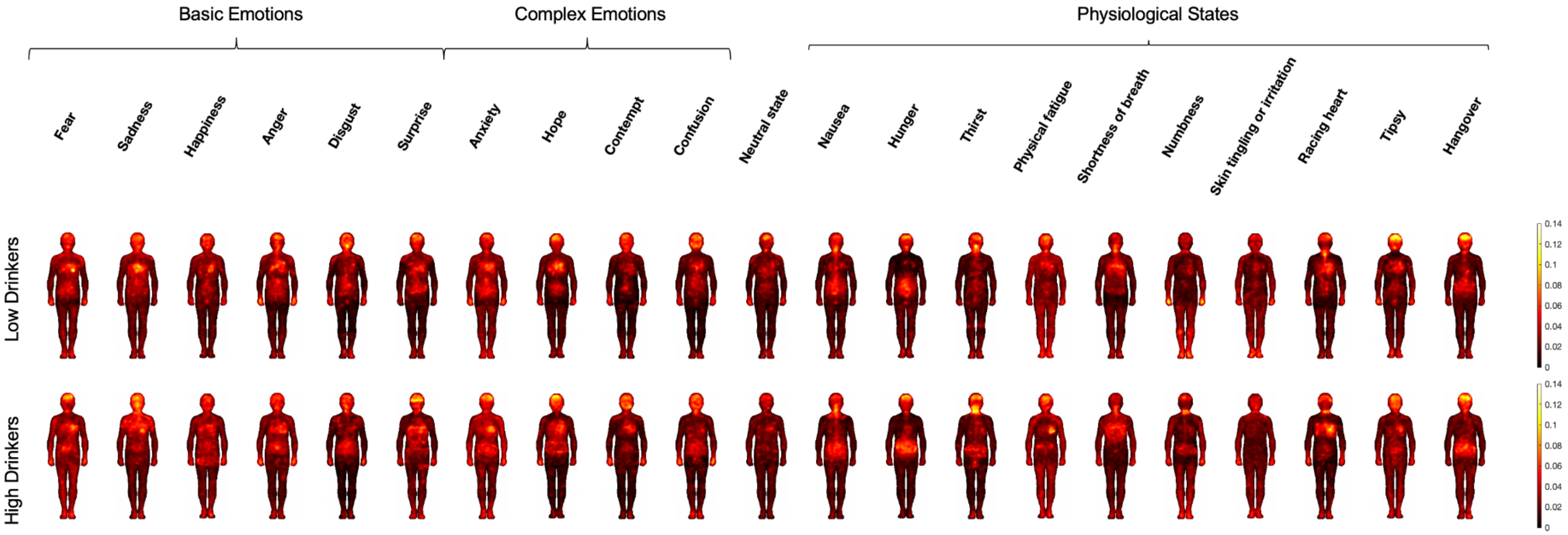

Fig S4. Standard deviation maps for the bodily sensations of Low and High Drinkers.

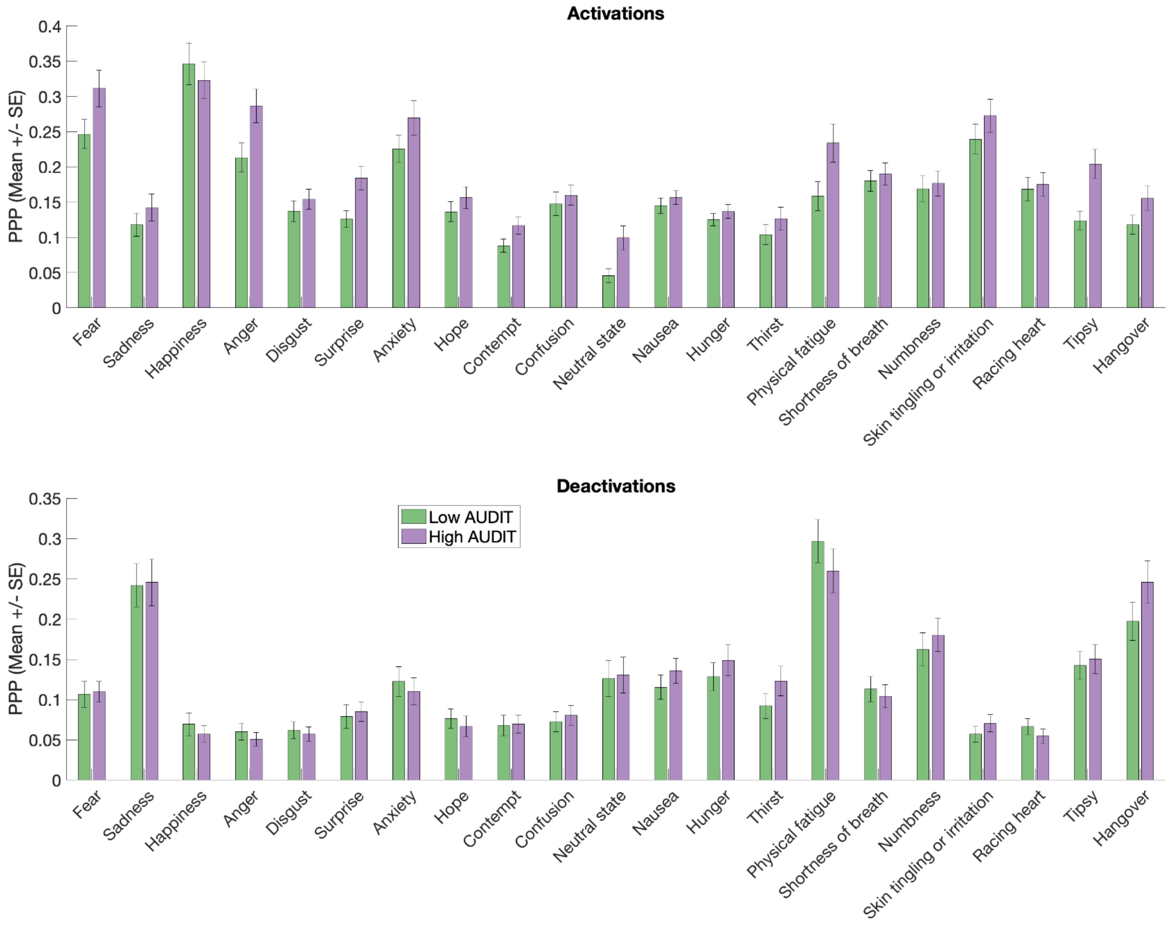

Fig S5. The proportion of pixels painted for each BSM and group. Error bars represent the standard error of the mean.

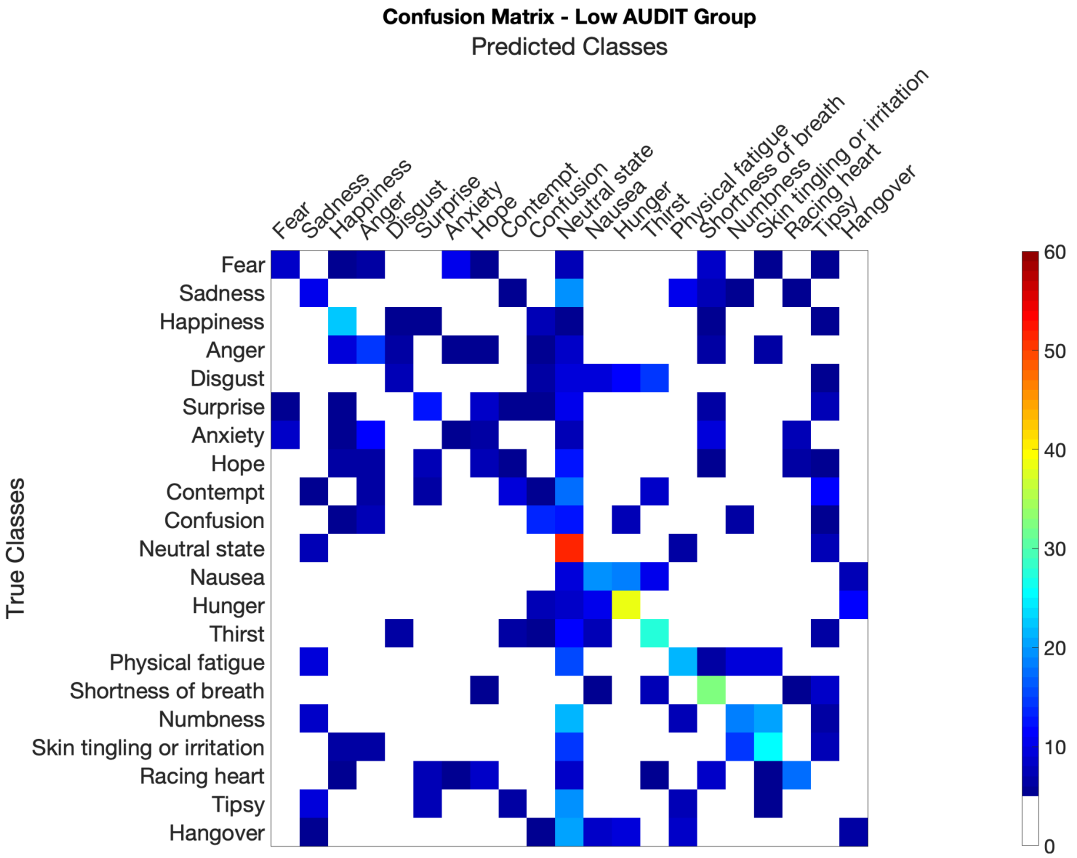
Confusion Matrix - Low AUDIT Group
Predicted Classes
Fear
Sadness
Happiness
Anger
Disgust
Surprise
Anxiety
Hope
Contempt
Confusion
Neutral state
Nausea
Hunger
Thirst
Physical fatigue
Shortness of breath
Numbness
Skin tingling or irritation
Racing heart
Tipsy
Hangover
True Classes
0
10
20
30
40
50
60

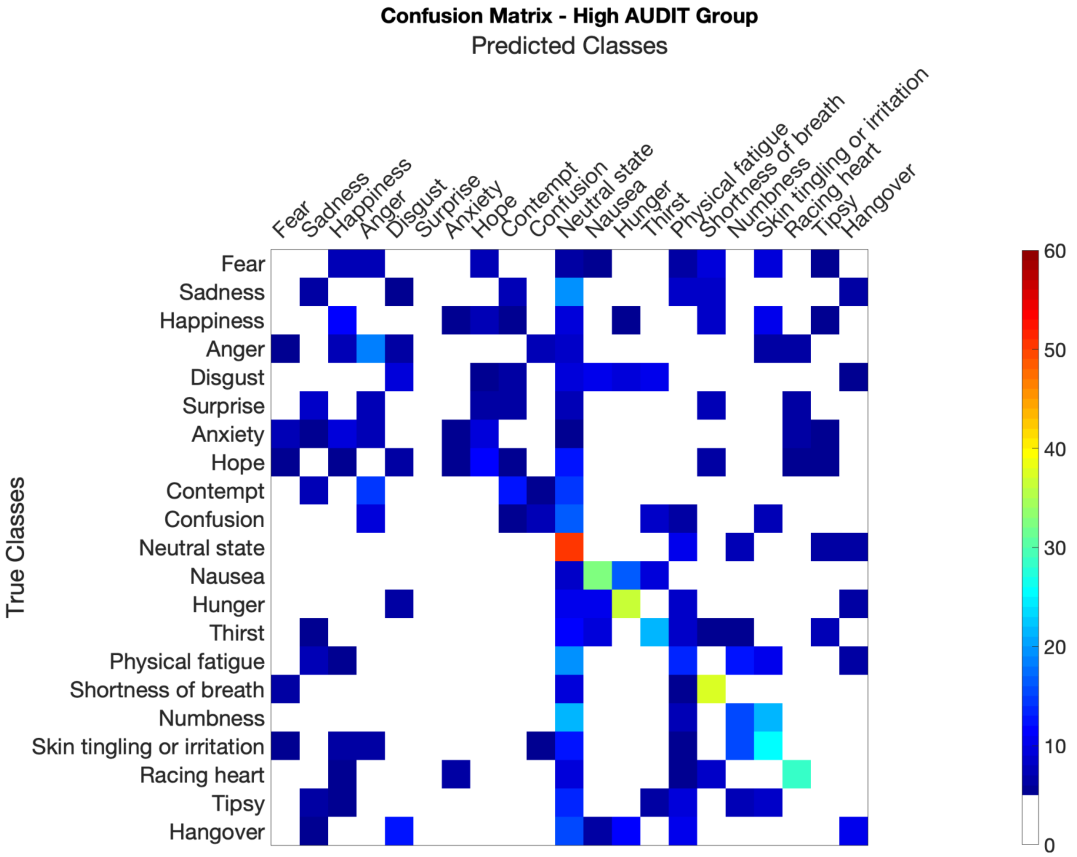
Confusion Matrix - High AUDIT Group
Predicted Classes
Fear
Sadness
Happiness
Anger
Disgust
Surprise
Anxiety
Hope
Contempt
Confusion
Neutral state
Nausea
Hunger
Thirst
Physical fatigue
Shortness of breath
Numbness
Skin tingling or irritation
Racing heart
Tipsy
Hangover
True Classes
0
10
20
30
40
50
60

Fig S6. Confusion matrices for the complete classification scheme for low and high drinking groups between predicted and true classifications. Classifications below the chance level (5%) are depicted in white. Colour bars represent classification accuracies in %.
